# Epidemiology, Diagnosis, and Prevention of Sparganosis in Asia

**DOI:** 10.3390/ani12121578

**Published:** 2022-06-18

**Authors:** Wei Liu, Tengfang Gong, Shuyu Chen, Quan Liu, Haoying Zhou, Junlin He, Yong Wu, Fen Li, Yisong Liu

**Affiliations:** 1 Research Center for Parasites & Vectors, College of Veterinary Medicine, Hunan Agricultural University, Changsha 410128, China; weiliupro@hunau.edu.cn (W.L.); gongtf@stu.hunau.edu.cn (T.G.); ShuyuChen2021@stu.hunau.edu.cn (S.C.); zhy20021006111111@163.com (H.Z.); hejunlin607@163.com (J.H.); 2 Hunan Provincial the Key Laboratory of Protein Engineering in Animal Vaccine, College of Veterinary Medicine, Hunan Agricultural University, Changsha 410128, China; wuyong712@126.com; 3 School of Life Sciences and Engineering, Foshan University, Foshan 528225, China; liuquan@fosu.edu.cn

**Keywords:** sparganum, epidemiological investigation, molecular diagnosis

## Abstract

**Simple Summary:**

This study provides information on the life cycle, clinical characteristics, pathogenesis, and molecular diagnosis and describes the geographical distribution and infection characteristics of *Spirometra* in the host, which are of considerable significance to preventing *Spirometra* parasites.

**Abstract:**

Sparganosis is a zoonotic parasitic disease caused by the larvae (spargana) of the genus *Spirometra*, which is widely distributed globally and threatens human health. More than 60 species of *Spirometra* have already been identified, and over 2000 cases have been reported. This review summarizes the prevalence of humans, frogs, snakes, and other animals with spargana. Furthermore, the infection mode, distribution, and site are summarized and analyzed. We also describe the epidemiology, molecular diagnosis, and other aspects which are of considerable significance to preventing sparganum.

## 1. Introduction

*Spirometra* tapeworm, belonging to the *Diphyllobothriidae* family, is one of the most harmful foodborne parasites in the world [1,2]. A total of more than 60 species of *Spirometra* have been reported worldwide, but only four are considered valid (*S. erinaceieuropaei*, *S. mansonoides*, *S. pretoriensis*, *S. theileri*) [3]. There are two species recorded in China: *S. erinaceieuropaei* and *S. decipiens*. Identifying species is still quite confusing, and the larval stage is mainly parasitic in animals such as frogs and snakes [3]. Generally, all spargana detected from mammals can develop into adult *Spirometra* worms in the intestines of dogs and cats [2]. In the Americas, the life history and morphology of *S. mansonoides* are similar to those of *S. erinaceieuropaei*. Many scholars believe that the two are the same species, but their opinions have not unified [4]. 

After infecting humans, spargana migrate to various tissues and organs, such as subcutaneous muscles, the liver, the lungs, and the brain, resulting in local tissue damage and paralysis, blindness, and even death [5]. Sparganosis has a worldwide distribution and is mainly found in China, Japan, South Korea, Thailand, and Southeast Asian countries [6,7]. Sporadic cases occur in South America, Europe, and Africa [8]. Many infections are asymptomatic. Only the more overt or severe cases are diagnosed, such as those with fever and neurological symptoms that mainly appear as seizures, light hemiplegia, progressive headache, and disturbance of consciousness [9,10,11]. Due to the misunderstanding of folk prescriptions in some remote rural areas in China, people apply fresh snake and frog meat to wounds or abscesses to relieve skin ulcers and eye inflammation [12], even swallowing raw or semi-raw frog and snake meat. Therefore, frogs and snakes become the primary source of infection [13]. 

## 2. Lifecycle

**Figure 1:**
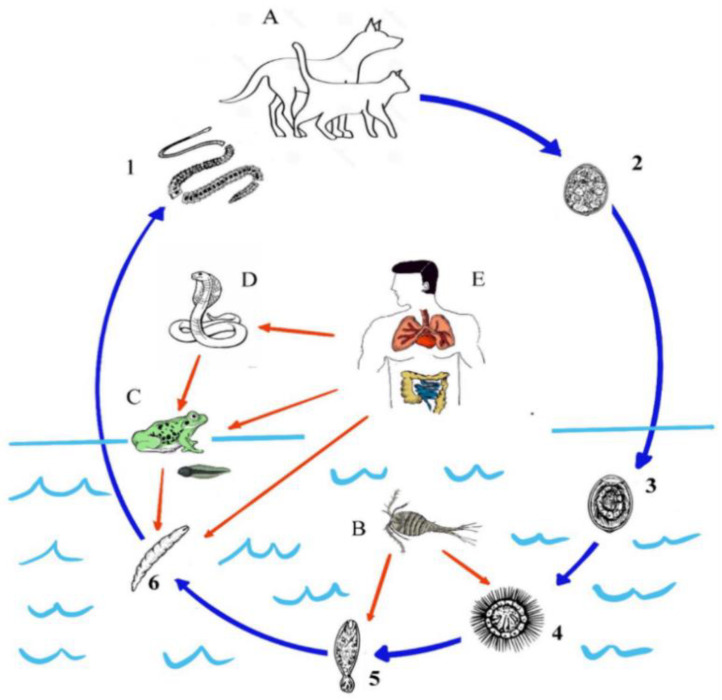
The life cycle of *Spirometra* tapeworm. 1: *Spirometra* adult worms; 2: eggs; 3: eggs hatch in water; 4: coracidia; 5: procercoid larvae; 6: sparganum: A: the definitive hosts: dogs and cats; B: the first intermediate hosts: cyclops; C: the second intermediate hosts: frogs and tadpoles; D, E: the paratenic hosts: human beings and snakes.

Most reports about *Spirometra* tapeworms worldwide are due to the disease caused by sparganum infection. The *Spirometra* tapeworm needs to live in two intermediate hosts and several paratenic hosts during the life cycle. Cyclops is the first host; snakes, frogs, fish, birds, and many other animals are the second intermediate host; and cats and dogs are the definitive hosts [14]. Sparganum can develop into adults in the intestines of cats and dogs. The eggs produced by the adults will be excreted in the host’s feces, and adults can be detected in the intestinal tract of the terminal host. Under the right conditions, the eggs can hatch into the coracidia, which, when eaten by the cyclops, enter the primary host and pass through the intestinal wall into the blood to develop into procercoid larvae. When the intermediate host, frog or fish, swallows the cyclops containing procercoid larvae, it will grow into the sparganum. When people, snakes, and other intermediates prey on hosts containing spargana, they will also be affected. However, spargana cannot develop into adults in these hosts and will migrate to many areas. Some of the most common parts are the legs and abdomen of frogs, the subcutaneous tissues and innards of snakes, and the brain and subcutaneous tissues of humans [7]. When a definitive host—a cat or dog—ingests a sparganum, it will develop into an adult in the intestinal tract. A new life cycle begins when the eggs are released from the body in the host’s feces [14] (Figure 1).

## 3. Pathogenesis 

While *Spirometra* adult worms lead a relatively settled life in the intestine of the definitive hosts, the spargana actively penetrate the intestinal wall and migrate to various tissues and organs, during which they must overcome several hurdles (e.g., host physical barrier, innate and acquired immune responses) before reaching their predilection sites. Soft tissues under the skin and in the brain parenchyma are particularly vulnerable to mechanical damage triggered by the invasive spargana, as reflected by the relatively common occurrence of subcutaneous and cerebral sparganosis [14]. Proliferative spargana (*S. proliferum*) demonstrates the capacity to reproduce asexually, branching and breaking down into numerous segments, facilitating their spread to other sites. Hydrolyzing enzymes generated by the plerocercoids contribute to further damage to host tissues [15]. 

Spargana produce several proteins that help invade host tissues and evade host immune surveillance. For example, spargana release a protein called sparganum growth factor (SGF) or “plerocercoid growth factor” (PGF) that is transported by the blood and interacts with growth hormone receptors [16]. Additionally, *S. erinaceieuropaei* generates a stage-specific cysteine protease of 36 kDa in the tegument and parenchymal tissue of plerocercoids but not in the adult worm or eggs. This protein induces a Th2-predominant immune response and enhances the survival of plerocercoids in the host [17]. Furthermore, *S. mansoni* secretes three neutral serine proteases (including two trypsin-like proteases of 198 and 104 kDa and one chymotrypsin-like serine protease of 36 kDa). Serine proteases are noted for possessing a nucleophilic serine residue at the active site, which mediates protein hydrolysis. The 198 and 104 kDa serine proteases of *S. mansoni* display collagenolytic activities involved in plerocercoid invasion and migration, whereas the 36 kDa serine protease can cleave human recombinant interferon-γ and bovine myelin essential protein and plays a role in immune evasion [18].

## 4. Clinical Features 

Spargana can invade various tissues (e.g., the brain, eyes, spinal cord, and breast and subcutaneous tissues) and induce local tissue damage, blindness, paralysis, and possible death. Human infection with *S. proliferum*, the larval form of an unspecified *Spirometra* species, involves multiple sites and organs, commonly called proliferative sparganosis. On the other hand, human infection with the larvae of other *Spirometra* species affects only one and no more than a few sites, known as nonproliferative sparganosis.

### 4.1. Proliferative Sparganosis

Resulting from infection with *S. proliferum*, the larval stage of a yet-to-be-identified *Spirometra* species, proliferative sparganosis is characterized by the invasion of numerous asexually multiplying plerocercoids into various tissues and organs, sometimes with fatal consequences. This disseminating disease occurs sporadically in Japan, Taiwan, and Thailand and rarely in Paraguay, Venezuela, and the USA [19].

**Figure 2:**
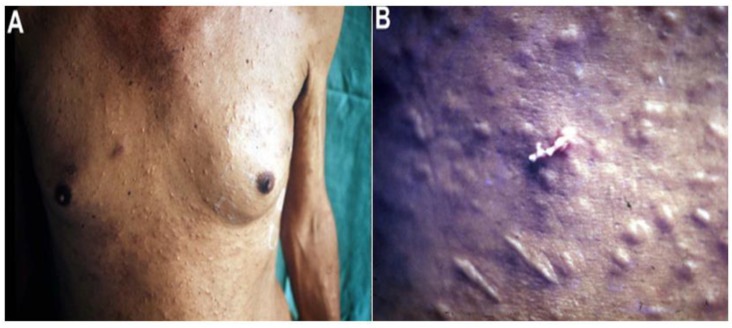
Proliferative sparganosis in a patient showing (**A**) dermal nodules on the chest wall and (**B**) *Sparganum* *proliferum* plerocercoid squeezing out of the skin. (This figure is from Kikuchi et al., [15]).

In cutaneous proliferative sparganosis, patients experience localized skin eruption, which then spreads to larger areas of skin as the parasite invades and migrates within the dermis, with a fatality rate of 87% (Figure 2) [15]. In internal proliferative sparganosis, patients develop nodules (or masses) in deep connective tissues or internal organs with no apparent dermal involvement, contributing to a 30% fatality rate. Many asexual larvae invade various tissues and organs, affecting the normal and nutritional function of tissues and organs, which may be the main reason for the poor prognosis [15].

Proliferative sparganosis in immunocompromised patients (due to AIDS, cancer, or allograft recipients) often has severe outcomes.

### 4.2. Nonproliferative Sparganosis

Due mainly to infection with *S. erinaceieuropaei* and—less frequently—*S. decipiens* in China, Japan, Korea, Taiwan, and Thailand and *S. mansonoides* in the USA, nonproliferative sparganosis is characterized by one or several plerocercoids that migrate in the connective tissues (and occasionally the lungs, liver, eyes, and central nervous system), producing simple mass effects. 

Subcutaneous sparganosis accounts for about 30% of clinical cases and often presents with slowly growing, rubbery, subcutaneous nodules, which may be painless (58%) or painful (24%) infected sites (14%) and hemorrhagic mass (3%) in the lower extremities (35%), trunk (26%), breast (17%), scrotum (11%), inguinal area (2%), upper extremities (2%), axilla (2%), penile shaft (2%), neck (2%), vulva (2%), and spermatic cord (1%) (Figure 2). The affected patients are mainly in the sixth decade of life (range 4–85 years) [20]. 

Accounting for about 25% of clinical cases, cerebral sparganosis involving the CNS manifests as seizure (20%), headache (12%), altered mental functions (e.g., confusion, memory loss; 11%), hemiparesis (6.8%), motor weakness (4%), cerebral hemorrhage, and fatigue; that involving the spinal cord exhibits voiding difficulty (38%), recurrent back pain (38%), and paresis (23%); and that involving both the CNS and spinal cord causes seizure, convulsion, increased intracranial pressure, facial palsy, hearing loss, voiding difficulty, and paresis of the lower extremities. Cerebral sparganosis appears to affect more men (76%) than women (24%), and infected patients are often in their thirties (20%), forties (23%), fifties (20%), and sixties (20%) [21,22].

Visceral sparganosis affecting the breast and abdomen accounts for about 26% of cases and is mainly attributed to swallowing live tadpoles or snake sacs to relieve fever. Clinical symptoms include dyspnea, chest pain, pleural effusion, empyema, intestinal obstruction, perforation, abscess formation on the bladder wall, and epididymis and penile shaft infections [23,24,25,26,27]. A male predominance (male: female ratio of 4.8:1) is observed among patients (mean age of 50 years; range 23–70 years). 

Ocular sparganosis constitutes about 13% of cases and is mainly due to the application of a traditional poultice of frog or snake flesh to subside sores or edema of inflamed eyes, carbuncle sores, or open wounds, which is popular in Southeast Asian countries (including southern China). It often causes painless mass with/without itching in the subconjunctival (68%), eyelid (25%), and intraorbital (6%) areas, as well as swelling, eyelid redness, excessive lacrimation, itching, or pain [28,29]. There is a male predilection (male:female ratio of 3:1) among patients (range 11–67 years). 

Muscular sparganosis induces poorly defined intramuscular mass with heterogeneous echotextures in the subcutaneous tissues and muscles and affects more males than females (male: female ratio of 3:1), with a mean age of 61 years among patients.

Systemic sparganosis is due to infection with multiple worms and often shows palpable subcutaneous masses/nodules in the breast, elbow, chest walls, abdominal wall, thighs, calf, and inguinal suprapubic areas.

## 5. Epidemiology 

### 5.1. S. erinaceieuropaei Infection 

**Figure 3:**
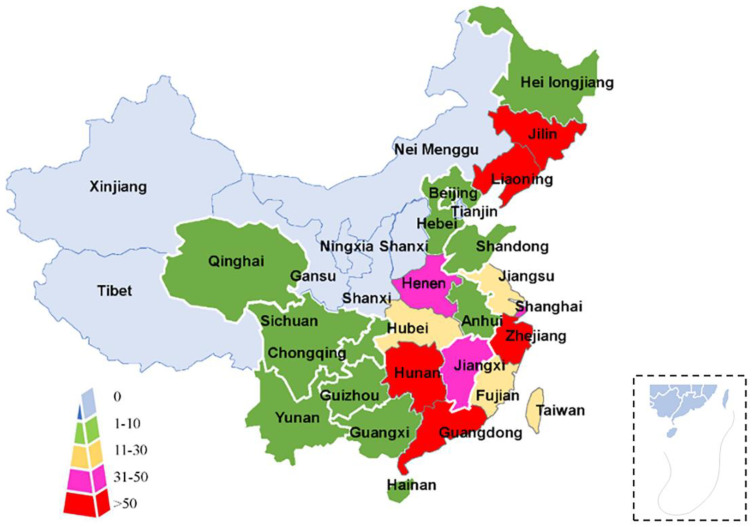
Geographical distribution of human sparganosis in mainland China.

***Human sparganosis***. When humans are infected, plerocercoid larvae migrate to subcutaneous tissues and other locations, forming a nodular mass or cyst and causing nonspecific discomfort, seizures, and headaches. The severity of larvae is due to the migration of the larvae and their place of residence. According to its clinical characteristics, sparganosis can be divided into sparganosis of the eye, sparganosis of the central nervous system, scrotal sclerosis of the viscera, subcutaneous sparganosis, and sparganosis of the oral and maxillofacial region [30]. China has reported over 1600 cases of human sparganosis, accounting for more than 80% of patients reported worldwide [14] (Figure 3).

In China, the first reported case of sparganosis occurred in Xiamen, Fujian Province, in 1882. From 1949 to 2014, 1359 cases of sparganosis infection were distributed in 27 provinces (municipalities/autonomous regions) in the country and were more common in the south and east provinces [31]. According to the reported literature from CNKI, our statistics are based on the declared area and the site of infection. According to Liu et al. [14], there were 146 cases from 2015 to 2019, with 118 cases involving the brain, one case involving the eyes, 16 cases involving the breasts and abdomen, six cases involving the lung, and one case involving the limbs. There were two cases of sparganosis in genital and urethral sites, one case in serum, and one case in thyroid infection. In the above statistics, there were 79 cases of sparganosis in Guangdong Province, ranking first, followed by Beijing with 24 patients and Hunan in third place (Table 1).

**Figure 4:**
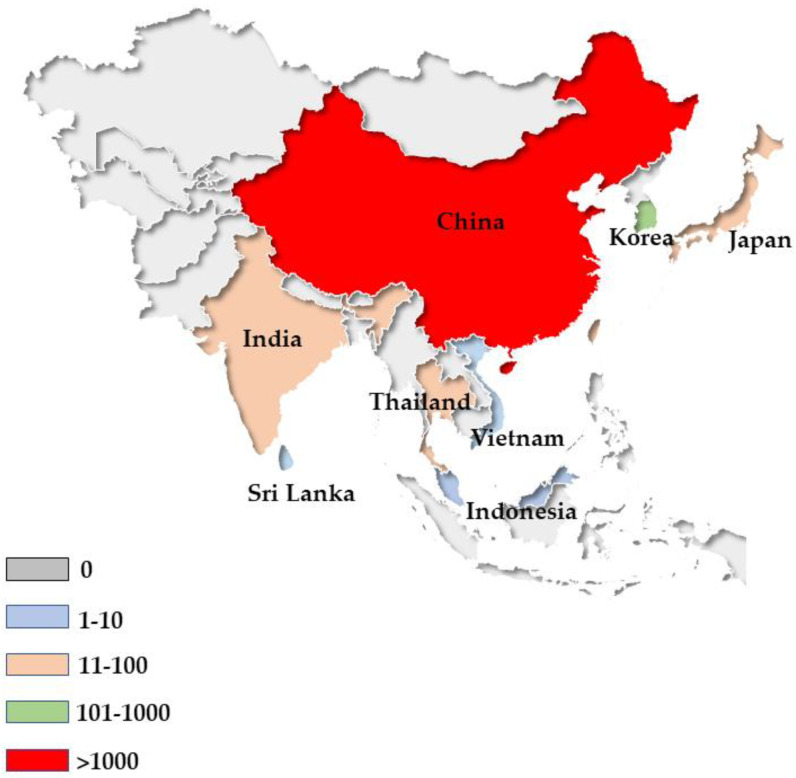
Geographical distribution of human sparganosis in Asia.

In addition to mainland China, there are also quite a few reports of people infected worldwide (Figure 4). In Asia, there are 24 cases in Taiwan [60,61], 32 cases in Japan [62,63,64,65,66], 63 cases in Thailand [5,64,67], and 438 cases in South Korea [7,68,69,70,71]. Though Asia has the highest number of infections, we can also find some infected reports on other continents. For example, there are 62 cases reported in the United States [62,72] and 18 cases in Europe [72]. 

***Animal sparganosis.*** There are 19 species of Cyclops, the first intermediate host of *Spirometra* tapeworms [73]. Frogs are the second intermediate host, and at least 14 species can be affected. Various vertebrates, such as snakes, birds, and pigs can be used as paratenic hosts.

**Frogs.** In China, 13 frog species have been infected with spargana [1]. The infection rates in different regions are significantly different for its crucial intermediate host frog. The *Rana nigromaculata* is the most systematically studied frog species in the epidemiological investigation of the main frog sparganosis. The existing literature from 1990 to 1999 counted the main statistics in three regions in China. The infection rates were 77.03% (Jiangxi province), 20.40% (Zhejiang province), and 12.25% (Sichuan province), and the highest infection intensity was 25. Sparganum infections were mainly parasitic on the thigh (91%), with a few on the back and head [74,75,76]. Statistics from 24 provinces from 2000 to 2011 included statistics on the infection of seven species of frogs by Li et al. The prevalence in frogs was on average 22.29%, ranging from 3.20% to 90.90%. The highest infection intensity was 77.60% [6]. Between 2011 and 2012, the sparganum infection statistics of frogs from two provinces of China were 29.4% and 26.3%, and the infection intensity was between 1 and 15 [77,78]. From June 2013 to August 2013, investigations were conducted on frog infections in different regions of Guangxi, and the infection rate was 30.7% [79]. From July 2013 to September 2018, through a large-scale investigation of frog infections in 34 provinces, autonomous regions, and municipalities in China, eight species of frogs were infected with spargana. The most common infection was *R. nigromaculata*, with a high infection rate of 14.1%. The subcutaneous muscle infection rate was the highest (68.1%), followed by the back at 14.94% and the abdomen at 9.98% [80]. The infection rate of frogs varies significantly by region, season, and type of frog [78,81,82,83,84].

Based on the above survey data, sparganum prevalence in frogs is relatively high. Sparganum infection frogs is mainly parasitic in the hind legs, followed by the chest, abdomen, and back [80]. In some areas of China, people apply raw frogs to wounds, eat raw tadpoles, or eat undercooked frogs, and the fresh legs of frogs are a central food. Therefore, the high parasitic rate increases the rate of sparganum infection. Wild frogs keep a high infection rate of *S. erinaceieuropaei* for a long period of time, which is an essential source of infection for human sparganosis. 

**Snake**. Snakes are intermediate hosts. They use frogs and other animals as food. Once having eaten frogs infected with spargana, snakes may become infected. Snakes infected with a small amount of sparganum do not show obvious symptoms, making it difficult for people to judge whether the snake carries the sparganum, increasing the risk of human infection. 

It has been reported in the literature that the spargana were found in approximately 20 species of snakes, and the infection rates of different snake species or in other regions were significantly different [30,85,86]. From 1990 to 2018 in China, the infection rate of spargana in Guizhou, Guangdong, Guangxi, Hunan, and other places was 22.2–100%, and the infection intensity was 100%. The favorable rates of spargana of the snakes in Jiangsu, Hunan, and Shanghai were above 90% (Table 2). In the early investigation of 75 snakes in different regions of Korea, the infection rate was 41% [87].

Additionally, we have counted spargana infection in snakes, such as the *Elaphe carinata*, *Zaocys* *dhumnade, Elaphe taeniura*, and *Ptyas kouros,* in nine provinces. The infection rate ranges from 0% to 99.8%, with an average infection rate of about 55.2%. The infection intensity ranges from 1 to 371. Spargana infection in frogs mainly parasitizes in the muscle and subcutaneous region, and a small part is parasitizes the body cavity. The epidemiological investigation of spargana helps to understand the spread of sparganosis and lays a foundation for the study of pathogenic characteristics, prevention, and treatment of sparganosis, which has important guiding significance (Table 3).

**Other animals**. In addition to critical intermediate hosts—frogs and snakes—other animals have been infected, such as cats, dogs, red foxes, ducks, and other species, but the related literature is scarce. There are many reports of dogs and cats being infected with adult *Spirometra* worms, and the infection rates range from 1% to 33% (dog), and 0.9% to 83% (cat) [4,97,98]. Cats and dogs are the most common pets in our lives. People are in close contact with these two animals, which increases the chance of spargana infection in humans and animals. Hygienic management and regular inspections of cats and dogs are also an aspect of reducing sparganosis incidence. In 2013, a survey found that 147 red foxes in Australia [99] contain many parasites, including adult *Spirometra* worms, with an infection rate of 5.4%. In 2013, Mauritius found sparganum in seven macaque monkeys [100]. Spargana also parasitized hedgehogs in Guizhou in 1959, and spargana in Hubei have also been reported to be parasitic in hedgehogs [101], making the animals living in our surroundings more likely to develop scabies, so education on personal hygiene and sparganosis prevention should be prioritized.

### 5.2. Spirometra Decipiens Infection

Much molecular identification of *S. erinaceieuropaei* and *S. decipiens* in the world came to two different species of the same genus [3,102,103,104,105]. However, there are few reports of *S. erinaceieuropaei* and *S. decipiens* infection, mainly in Korea. Fifty cases of human sparganosis were reported in Korea in 2009, which were caused by *S. decipiens* infection [103]. Fourteen *S. decipiens* infections were reported in South Korea from 1973 to 2008, occurring in three areas of Jinju, Seoul, and Chuncheon—eight in cats, two in snakes, two in dogs, and one in ducks [106]. Korea reported an in vivo parasite detection report on stray cats by cytochrome c oxidase subunit 1 (*cox*1) contrast sequences identified as *S. decipiens* parasitism (Table 4).

## 6. Diagnosis

### 6.1. Clinical Diagnosis

Clinical diagnosis of sparganosis involves a physical examination of cutaneous and other nodules, computed tomography (CT) assessment of the nodule location and dimension (typically hypo or isodense images without the tunnel sign), and magnetic resonance imaging (MRI) demonstration of a mass lesion or vasculopathy (typically hypointense and hyperintense lesions on T1- and T2-weighted images, with variable enhancement, together with possible tunnel sign-on postcontrast images). More specifically, cerebral sparganosis on CT reveals unilateral involvement, low-density lesions in the white matter with ipsilateral ventricular dilatation and localized cortical atrophy, nodular or irregular enhancement with spotty calcification, and changes in the location of lesions, suggesting long-term inflammation with active granuloma and irreversible brain damage due to worm migration and the histotoxic effects of proteases secreted by the worm. Despite their obvious value, these procedures lack specificity and do not give a definitive diagnosis [109,110].

### 6.2. Laboratory Diagnosis

Laboratory methods for diagnosing sparganosis include macro- and microscopic identification of spargana recovered through tissue biopsy, biochemical tests, serological assays, and molecular techniques.

Macroscopically, spargana appear as a thin, white solid flatworm of a few millimeters to several centimeters in length, covered by a gliotic wall and inflammatory exudates. Microscopically, spargana display a solid noncavitated body without a bladder wall, hooked scolex, or suckers, unlike other cysticerci. The histological identification of *Spirometra* is based on finding a characteristic deep-folded tegument and calcareous corpuscles.

Biochemical tests may show pronounced peripheral eosinophilia but not CSF eosinophilia [111,112]. 

Serological assays (e.g., ELISA) offer an alternative approach to the diagnosis of sparganosis, especially when spargana are invisible (e.g., in the case of pleural sparganosis) or if biopsy or removal of spargana is impractical [113]. Indeed, using recombinant cysteine protease from sparganum plerocercoids as antigen, a sensitivity of 100% and a specificity of 98.22% are achieved in ELISA to diagnose human sparganosis. 

### 6.3. Molecular Diagnosis

With the development of modern molecular biology techniques, the selection of appropriate molecular markers can identify and analyze parasites accurately. Mitochondria have a small-genome matrilineal inheritance and are not subject to genome recombination ectopic effects, so they are very suitable for studying intraspecies genetic variation and molecular diagnosis. In recent years, many studies in China and abroad have used different mitochondria and ribosomal marker molecules to conduct molecular identification and genetic variation studies on isolates of spargana from various host sources.

The mitochondrial genome has a simple structure, maternal inheritance, lack of recombination, and fast evolution rate. It is particularly suitable as a marker for genetic research. Accurate classification, variants, and evolutionary history are important [114]. According to the study, the sensitivity of the *cox*1 development tree is higher than that of the ITS sequence, and the evolutionary distance of the tree is substantially lower than that of the ITS sequence. The molecular phylogenetic relationship of *S. erinaceieuropae* can be deduced from this. ITS gene sequence may be more suitable for molecular markers for breeding, and *cox*1 is ideal for studying genetic polymorphisms within species [50].

Since 2010, our team has carried out a series of molecular biology studies on adult or larval isolates of different host *Spirometra* tapeworms in Hunan province and made research progress. We mainly used mitochondrial *cox*1, cytochrome c oxidase subunit 3 (*cox*3), nicotinamide adenine dinucleotide dehydrogenase subunit1(*nad*1), *nad*4, and *nad*5 to analyze the different ribosomal DNA internal transcribed spacer (ITS) gene sequence of Hunan province and investigate the developmental relationship between adult and larval isolates of *Spirometra* tapeworms from different host sources (e.g., dogs, snakes, and frogs) [82,95,114,115,116,117,118,119,120,121]. The main findings are as follows: (1) According to a molecular biology study, different hosts of parasitic *Spirometra* tapeworms are *S.* *erinaceieuropaei*, and the variation rate of individual genes of *S. erinaceieuropaei* in different regions of Hunan province was low; (2) There are some differences in the various rates of different gene sequences, but in general, the variation rates of different mitochondrial genes are lower than those of ribosome ITS gene sequences, and the various rates of mitochondrial *cox*1 and *cox*3 genes may be lower than those of mitochondrial *nad*1 and *nad*4 gene sequences [114,119]; (3) However, compared with other *Diphyllobothriidae* tapeworms, *S. erinaceieuropaei* is the closest relative to the genus of spargana. Our findings have been confirmed by many studies [38,109,114]. In addition, other studies have shown that the molecular identification of human sparganum is *S. erinaceieuropaei*, which ensures *S. erinaceieuropaei* is a zoonosis once more. The study of the population evolution of the disease is critical [13,63,64,67] (Table 5).

*S. erinaceieuropaei* has unique biological characteristics, resulting in different *Spirometra* reproductive isolation phenomena between isolates; reproductive isolation, which leads to additional genetic material not establishing communication between individuals; and genetic variation being easily affected by climate, environment, and geography. The steady accumulation of genetic variation may be a large regional or environmental climate difference between strains, causing more significant differences in genetic material or evolution. Therefore, it is vital to analyze the genetic variation of adult or larval isolates of *S. erinaceieuropaei* in geographical regions to understand the evolution or origin of the isolates. The phylogeny of sparganum isolates from frogs collected from the different areas of China was studied using other molecular markers (mitochondrial *cox*1, *cyt*b). The phylogenetic tree shows that spargana from different regions can be divided into two branches. The sources of sparganum from Henan, Hunan, Anhui, Zhejiang, Jiangsu, and Chongqing are located in one branch. The spargana of Yunnan, Hainan, and Guangxi frogs are isolated in another branch. This may be related to the environment, such as geographical differences. In addition, the results of software analysis show that although the two groups may have started to differentiate in the middle Pliocene, their differentiation time is also different [2,123,124].

In addition, Polish researchers constructed the host germline development relationship of *S. erinaceieuropaei* isolates from different regions of the world with the mitochondrial *cox*1 gene sequence. They found no significant differences between isolates from different host sources. Still, there are substantial differences between isolates belonging to different area branches: the Asian (China, Laos, Myanmar, and Japan and other countries) and additional Australian sources that host the sparganum are located in one branch, and the Poland region’s separation plant is located in another branch, which explains why strains in Europe, Asia, and other regions’ isolates’ genetic evolution are more considerably different. However, the evolution rates of sparganum isolates from different hosts in the same area differed slightly, which is highly consistent with our recent research results [95,127].

## 7. Control (Including Treatment and Prevention)

For most *S. erinaceieuropaei* infections, surgical removal of worms is the primary method. The treatment of sparganosis depends on the number of worms and the parasitic site. Those superficially located parasites can be removed by surgery under local anesthesia. Care should be taken during surgery to avoid worm body breakage in order to prevent the worms’ cephalic region sections from remaining and continuing to grow and cause recurrence. Once a worm’s body is broken, it can be removed entirely under local anesthesia with ether. If worms cannot be surgically removed, 40% ethanol novocaine can be injected into the induration to kill the cleft worm [75].

Spargana mainly infect through the mouth and wounds, so it is of great significance to publicize the prevention and treatment of sparganum. In some parts of China, ignorant superstitions are applied to raw frogs and snake meat, significantly increasing the chance of sparganosis infection. In recent years, more and more people are advocating that if they eat semi-cooked frogs, snakes, birds, and pigs with sparganum, they will become infected. In addition, humans can also be infected through the eyes when swimming [50]. In summary, there are many risks in our lives where we may be contaminated with sparganum. We must stay vigilant and eliminate bad habits.

## 8. Conclusions and Future Perspectives

*Spirometra* is a neglected human pathogen due to its relatively rare occurrence in humans and complex life cycles. It is still unknown why *sparganum* *proliferum* (the larval stage of an unknown *Spirometra* species) demonstrates a higher pathogenic potential than other human-infecting *Spirometra* *spp*. (including *S. erinaceieuropaei, S. mansoni, S. mansonoides, and S. decipiens*.) in causing proliferative sparganosis instead of nonproliferative sparganosis. Further research in these areas, such as more precise species identification and control of *spirometra* *spp*, will be necessary and potentially rewarding. 

## Figures and Tables

**Table 1:** Parasitic location of human sparganosis in China (2015–2021).

Province	Brain and Spine	Eye	Breast and Abdomen	Lung	Limbs	Other Positions	References
Anhui	1		1				[32,33]
Hunan	9		3	2		1(Ovary)	[34]
Zhejiang	1		4	2	1	1(Serum)	[12,35,36,37,38,39,40,41]
Hubei	2		4	2		1(Thyroid)	[42,43,44,45,46,47,48,49]
Jiangxi			2			1(Urethra)	[50,51,52]
Chongqing	2				1		[53,54]
Guangdong	79						[55]
Guangxi			1				[56]
Jilin		1	1				[57,58]
Beijing	24						[59]
Total	118	1	16	6	2	4	

**Table 2:** Epidemiological investigation of snake sparganosis in China (1990–2018).

Province	No. Positive/No. Tested (Prevalence, %)	Intensity of Infection	References
Guizhou	47/204 (23)	1–125	[83,88]
Jiangsu	3/3 (100.00)	2–99	[89]
Guangxi	35/158 (22.2) 3/6 (50)	1–208	[79,86]
Guangdong	62/177 (35)	1–44	[85,90]
Sichuan	5/16 (31.3)	1–371	[91]
Shanghai	45/49 (91.8)	1–294	[30,92]
Zhejiang	5/6 (83.3) 5/10 (50.0)	1–143	[93,94]
Hunan	344/375 (91.7)	1–70	[95]
Jilin	134/435 (30.8)	—	[96]

**Table 3:** Infection investigation of different species of snake.

Species	No. Positive/No. Tested (Prevalence, %)	Intensity of Infection
*Elaphe carinata*	91/189 (48.1)	1~294
*Elaphe mandarins*	0/2 (0)	0
*Zaocys dhumnades*	25/45 (55.5)	1~371
*Elaphe taeniura*	8/37 (21.6)	1~125
*Ptyas korros*	15/49 (30.6)	1~125
*Elaphe radiata*	1/21 (4.8)	1~125
*Naja atra*	320/363 (55)	1~208
*Ptyas mucosus*	36/114 (31.5)	1~208
*Lycodon rufozonnatum*	31/53 (58.4)	0~44
*Bungarus multicinctus*	7/48 (14.6)	0~44
*Bungarus fasciatus*	2/22 (9.1)	1~43
*Tryptelytrops albolabris*	1/4 (25)	1~63
*Protobothrops jerdonii*	3/5 (60)	1~67
Other Species	0/4 (0)	0
Total	542/981 (55.2)	1~371

**Table 4:** *S.decipiens* molecular diagnosis.

Genes	Primers	Sequence (5′-3′)	References
*cox*1	spcox1f	5′-GTA TTG AAG GAA TTA GTT AGG TTA-3′	[104]
	spcox1r	5′-CAA CCC AAT TAA ATT AAG TTC CAC-3′	
*cox*1	Se/Sd-7963F	5ʹ-ACG TGG TTT GTG GTG GCT CAT TTT-3ʹ	[103]
	Sd8584R	5ʹ-GTA TCA AGT TGG TTA GGA AGT TAA-3ʹ	
*cox*1	p1f	5′-TGG TTT TTT GGA CAT CCT GAA -3′	[107]
	p1r	5′-ATC ACA TAA TGA AAG TGA GCC-3′	
rRNA	rRNA F	5′GAT TTT GTA AAT CAG GGG GTA-3′	[107]
	rRNA R	5′-AAT TTA TGC GAT TCA CCT TAA-3′	
*nad*4	Se/Sd-1800F	5′-TAT TTT CGG TTG GTG CTG TAG-3′	[108]
	Sd-2317R	5′-TCC TCC CCC CAC ACG ACA AAA-3′	
lrDNA	Se/Sd-7955F	5′-ACG TGG TTT GTG GTG GCT CAT TTT-3′	[108]
	Sd-8567R	5′-TTA TTA ACT TCC TAA CCA ACT TGAT AC-3′	

**Table 5:** *S.erinaceieuropaei* molecular diagnosis.

Genes	Primers	Sequence (5′-3′)	References
*cox*1	JB3	5′-TTTTTTGGGCATCCTGAGGTTTAT-3′	[82]
	JB4.5	5′-AAAGAAAGAACATAATGAAAATG-3′	
*cox*1	JB3	5′-TTTTTTGGGCATCCTGAGGTTTAT-3′	[122]
	JB4.5	5′-AAAGAAAGAACATAATGAAAATG-3′	
*cox*1	JB3	5′-TTTTTTGGGCATCCTGAGGTTTAT-3′	[95]
	JB4.5	5′-AAAGAAAGAACATAATGAAAATG-3′	
*cox*1	Cox1-F	5′-TAGACTAAGTGTTTCAAAACACTA-3′	[123]
	Cox1-R	5′-ATAGCATGATCGAAAAGG-3′	
*cox*1	Cox1-F	5′-TAGACTAAGTGTTTCAAAACACTA-3′	[124]
	Cox1-R	5′-ATAGCATGATGCAAAAGG-3′	
*cox*1	Spi-CO1F	5′-GACTAAGTGTTTTCAAAACACTAAGTG-3′	[105]
	Spi-CO1R	5′-CAC CCT ACC CCT GAT TTA CAA AAT-3′	
*cox*1	Se658-F	5′-TTTGATCCTTTGGGTGGTGG-3′	[67]
	Se1124-R	5′-ACCACAAACCACGTGTCATG-3′	
*cox*1	cox1-F	5′-CGGCTTTTTTTGATCCTTTGGGTGG-3′	[64]
	cox1- R	5′-GTATCATATGAACAACCTAATTTAC-3′	
*cox*1	12STaen-aFF	5′-CAC AGT GCC AGC ATC YGC GGT-3′	[63]
	12STaeniaRR	5′-GAG GGT GAC GGG CGG TGT GTA C-3′	
*cox*1	JB3	5′-TTTTTTGGGCATCCTGAGGTTTAT-3′	[64]
	JB4.5	5′-AAAGAAAGAACATAATGAAAATG-3′	
*cox*3	Secox3F	5′-GGGTGTCATTTCTTCCTATTTTTAA-3′	[120]
	Secox3R	5′-AAATGTCAATACCAAGTAACTAAAG-3′	
*cytb*	Cob-F	5′-TGATAGTATTAAACTGGC-3′	[123]
	Cob-R	5′-TCAACAGTTGAAACAACCA-3′	
*cytb*	Cob-F	5′-TGATAGTATTAAACTGGC-3′	[124]
	Cob-R	5′-TCAACAGTTGAAACAACCA-3′	
*nad*1	Nad1u	5′-ATAAGGTGGGGGTGATGGGGTTG-3′	[121]
	Nad1d	5′-ATAAAAAATAAAAGATGAAAGGG-3′	
*nad*1	Nad1u	5′-ATAAGGTGGGGGTGATGGGGTTG-3′	[121]
	Nad1d	5′-ATAAAAAATAAAAGATGAAAGGG-3′	
*nad*1	Spi-ND1F	5′-GGA GAATATTGGTTTGTCTAACCA-3′	[105]
	Spi-ND1R	5′-CCTTCTTAACGTTAACAGCATTAC GAT- 3′	
*pnad*1	Senad1F	5′-ATAAGGTGGGGGTGATGGGGTTG-3′	[120]
	Senad1R	5′-ATAAAAAATAAAAGATGAAAGGG-3′	
*nad*4	ND4F	5′-GAGTCTCCTTATTCTGAGCG-3′	[13]
	ND4R	5′-ATAGTAGTAGGAAATGAACA-3′	
*pnad*4	Senad4F	5′-TTTTTTCCTTGGGTTAAGATTAA-3′	[120]
	Senad4R	5′-GCTACTACCCTCAAAAGACTCAC-3′	
*nad*5	SCND5F	5′-TCATACTGGGTCTATCAGGTGTT-3′	[122]
	SCND5R	5′-ACAGCAAAGTTAGGGGGTAATAGGT-3′	
ITS	BD1	5′-GTCGTAACAAGGTTTCCG-3′	[125]
	BD2	5′-TATGCTTAAATTCAGCGGGT-3′	
ITS	NC5	5′-GTAGGTGAACCTGCGGAAGGATCATT-3′	[126]
	NC2	5′-TTAGTTTCTTTTCCTCCGCT-3′	
ITS	NC5	5′-GTAGGTGAACCTGCGGAAGGATCATT-3′	[119]
	NC2	5′-TTAGTTTCTTTTCCTCCGCT-3′	
ITS	NC5	5′-GTAGGTGAACCTGCGGAAGGATCATT-3′	[120]
	NC2	5′-TTAGTTTCTTTTCCTCCGCT-3′	
ITS	BD1	5′-GTCGTAACAAGGTTTCCG-3′	[13]
	BD2	5′-TATGCTTAAATTCAGCGGGT-3′	
28s	28S-F	5′-CACCGAAGC CTGCGGTA-3′	[63]
	28S-R	5′-GAAGGTCGACCTGGTGAA-3′	
*prrn*S	SCRRNSF	5′-TAGTTTGGCAGTGAGTTATTCCG-3′	[118]
	SCRRNSR	5′-GGCTACCTTGTTACGACT-TACCTCA-3′	

## Data Availability

Not applicable.
